# Histone H3 gene is not a suitable marker to distinguish *Alternaria tenuissima* from *A*. *alternata* affecting potato

**DOI:** 10.1371/journal.pone.0231961

**Published:** 2020-04-23

**Authors:** Yue Zhang, Peiyu Tian, Guohua Duan, Fangluan Gao, Guido Schnabel, Jiasui Zhan, Fengping Chen

**Affiliations:** 1 Fujian Key Laboratory of Plant Virology, Institute of Plant Virology, Fujian Agriculture and Forestry University, Fuzhou, China; 2 Department of Plant and Environmental Sciences, Clemson University, Clemson, SC, United States of America; 3 State Key Laboratory of Ecological Pest Control for Fujian and Taiwan Crops, Fujian Agriculture and Forestry University, Fuzhou, China; Wilfrid Laurier University, CANADA

## Abstract

Potato Alternaria leaf blight is one of the economically most important disease in potato production worldwide. A recent study reported a quick method to distinguish main *Alternaria* pathogens *A*. *tenuissima*, *A*. *alternata*, and *A*. *solani* using partial histone H3 gene sequences. Using this method, our collection of 79 isolates from 8 provinces in China were presumably separated into *A*. *tenussima* and *A*. *alternata*. But in depth morphological and genetic analysis casted doubt on this identification. Culture morphologies of six presumed *A*. *alternata* isolates (PresA_alt) and six presumed *A*. *tenuissima* isolates (PresA_ten) were not significantly different. PresA_ten isolates also produced conidia in branched chains which supposed to be *A*. *aternata*. Phylogenetic analyses were conducted using internal transcribed spacer region (ITS) and five genes commonly used for species identification including glyceraldehyde-3-phosphate dehydrogenase (*GPDH*), translation elongation factor 1-alpha (*TEF1*), β-tubulin, plasma membrane ATPase (*ATPase*), and calmodulin genes. The results showed that *GPDH* and *TEF1* sequences of PresA_alt and PresA_ten isolates were identical. The 12 isolates did not cluster by presumed species neither by individual or concatenated sequence comparisons. The phylogeny–trait association analysis confirmed that the two group isolates were undistinguishable by those molecular markers. Analysis of histone H3 gene sequences revealed variable intron sequences between PresA_ten and PresA_alt isolates, but the amino acid sequences were identical. Our results indicate that the previously published method to distinguish *Alternaria* species based on histone H3 gene sequence variation is inaccurate and that the prevalence of *A*. *tenuissima* isolates in China was likely overestimated.

## Introduction

Potato is the economically fourth most important food crop and one of the most important cash crops in the world. Global potato production increased from about 240 million tons in 1980 to 377 million tons in 2016 (Food and Agriculture Organization, 2017). But growing potatoes is challenging and marketable yield is threatened by many pests and diseases. Alternaria leaf blight is one of the major diseases in potato production, known as early blight and brown spot expressing similar symptoms in the early stage. Early blight is characterized by typical symptoms of concentric rings on leaves favored by high temperature and alternating periods of dry weather and high humidity [1]. The disease reduces yield, affects tuber size, and tuber quality [2]. Brown spot expresses small, irregular to circular lesions coalescing across large veins resulting in significant leaf damage [3].

The known Alternaria leaf blight pathogen is *Alternaria solani*, which was initially considered to be the causal agent of early blight in potato and other Solanaceae crops [4]. But various other *Alternaria* pathogens have also been identified. For example, *A*. *alternata*, *A*.*tenuissima*, *A*. *dumosa*, *A*. *arborescens* and *A*. *infectoria* have been reported in major potato growing regions in Iran [5]; *A*. *protenta* in Algeria [6]; *A*. *alternata*, *A*. *arborescens*, *A*. *protenta*, and *A*. *grandis* in Europe [7]; *A*. *longipes* in Pakistan [8]; *A*. *arborescens*, *A*. *alternata*, and *A*. *arbusti* in United States [9]; and *A*. *tenuissima* and *A*. *alternata* in China [10].

Morphologically, *Alternaria* species can be divided into large-spored and small-spored species. The primarily large-spored and small-spored pathogens are generally considered to be *A*. *solani* and *A*. *alternata* respectively, causing seriously threat in potato production although other species were reported recently [9,10]. Most *Alternaria* species of potato are small-spored; only *A*. *solani* and *A*. *grandis* are large-spored. Large-spored *A*. *tomatophila* was associated with early blight, but it is only weakly aggressive to potato [11].

Identification of *Alternaria* species is essential for disease management. Species can vary in fungicide sensitivity and in their ability to develop resistance to fungicides. For example, populations of *A*. *solani* and *A*. *alternata* differed in resistance to azoxystrobin in the Columbia Basin of Washington [12]. This phenomenon was also found in *Colletotrichum* complex [13–15]. Fungicides could also have different intrinsic activity in species complex, i.e less activity of benomyl was found in *C*. *acutatum* compared to *C*. *gloeosporioides* [16,17]. Another reason of importance to identify species is toxins production in some species. For example, *A*. *arborescens* produced AAL-toxin [18], but *A*. *infectoria* does not.

The distinction between small-spored *Alternaria* species has not been an easy task but progress seemed to have been made in recent years. Zheng et al. (2015) reported that *A*.*tenuissima* was successfully distinguished from *A*. *alternata* and *A*. *solani* based on the partial coding sequence of the histone H3 gene [10]. The three species revealed PCR amplicons of 546 bp, 440 bp and 489 bp in length, respectively. We used this method to screen our collection of isolates from various potato production regions in China, but noticed discrepancies and irregularities in results. The objective of this study was to investigate the accuracy of the method in distinguishing species by comparing assay results with morphological and in depth analysis of nucleotide sequences of key genes commonly used for fungal species separation.

## Material and methods

### Origin and collection of *Alternaria* isolates

A total of 79 single-spore isolates were obtained from 8 provinces in China including Yunnan, Fujian, Hebei, Inner Mongolia, Hubei, Henan, Heilongjiang and Shanxi during the 2011 to 2013 potato growing seasons (Table 1). Symptomatic leaves of circular lesion with concentric rings were collected from potato (one per plant) and leaf tissue (0.5 cm x 0.5 cm) from the demarcation zone of healthy and diseased areas was obtained. The pieces of tissue were rinsed in 75% alcohol for 2 min, then washed with sterile distilled water twice and dried in a laminar flow hood. The dried tissue was placed onto potato dextrose agar (PDA, including 200 g/l potato, 20 g/l glucose and 20 g/l agar) amended with 50 μg/ml streptomycin (Amresco, USA), and incubated at 25°C. After 3 days, actively growing mycelium was transferred to fresh PDA in petri dishes and incubated at 25°C until conidia were produced. Those were spread on water agar in a suspension and after 20 hrs of incubation at room temperature, and a single germinated conidium was isolated.

**Table 1:** Origin and frequency of *Alternaria* species from potato leaves identified by H3-1a/1b primer set.

Location	Collected Year	Isolate Designation	Subtotal	No. of isolates^z^		
				PresA_alt	PresA_ten	A_sol
Yunnan	2011	YN1-9	9	1	8	0
Fujian	2011	FJ1-8	8	6	2	0
Hebei	2012	HeB1-13	13	3	10	0
Inner Mongoria	2012	NMG1-12	12	0	12	0
Hubei	2012	HuB1-11	11	0	11	0
Henan	2012	HN1-8	8	0	8	0
Heilongjiang	2012	HLJ1-7	7	0	7	0
Shanxi	2013	SX1-11	11	1	10	0
Total number			79	11	68	0
Frequency of isolate (%)				13.9	86.1	0.0

^z^ PresA_alt, PresA_ten and A_sol were present presumed *A*. *alternata*, presumed *A*. *tenuissima* and *A*. *solani*, respectively.

### DNA extraction

Isolates were grown on PDA at 25°C in the dark for 7 days. Mycelium (~100 mg) was scraped off the medium surface, placed in a sterile, 2 mL centrifuge tube and lyophilized with a vacuum freeze dryer (Alpha1-2, Christ, Germany). The lyophilized mycelium was ground to powder with a mixer mill (MM400, Retsch, Germany). Genomic DNA was extracted from mycelium using a modified cetyltrimethylammonium bromide (CTAB) method [19]. Briefly, the mycelia powder was added to DNA extraction buffer (2% CTAB, 100 mM Tris-HCl [pH 8.0], 20 mM EDTA [pH8.0], and 1.4 M NaCl) and incubated for 30 min at 65°C. After extraction with one volume of phenol/chloroform/isoamyl alcohol (25:24:1), DNA was precipitated with one volume of isopropyl alcohol for 10 min at room temperature (23 ± 1°C). The suspension was centrifuged at 12,000 × g for 10 min and the pellet was washed with 75% alcohol and then 100% alcohol. DNA was dried in a heat block (OSE-100C, Tiangen Biotech, Beijng) and suspended in Tris-EDTA buffer (10 mM Tris-HCl and 1 Mm EDTA, pH 8.0).

### Identification of *Alternaria* species

All isolates were identified with H3-1a and H3-1b primers (S1 Table), which amplified partial coding sequences of histone H3 gene [10,20]. Polymerase chain reaction (PCR) was performed in a volume of 25 μl containing 1× PCR mix, 50 ng of DNA and 0.4 μM each primer (TransGen Biotech Co., Beijing) in an 2720 thermal cycler (Applied Biosystems, USA). The PCR was programed with an initial denaturation at 95°C for 4 min; 32 cycles of denaturation at 95°C for 30 s, annealing at 60°C for 40 s, extension at 72°C for 60s; and a final extension at 72°C for 7 min. PCR products were separated in 2.0% agarose gel in 1× Tris-acetate-EDTA buffer (40 mM Tris acetate and 1 mM EDTA, pH 8.0). A previous study indicated that the primers generated 546 bp, 489 bp and 440 bp amplicons, each corresponding to *A*.*tenuissima* (A_ten), *A*. *solani* (A_sol) and *A*. *alternata* (A_alt), respectively [10]. Six amplicons of each size were confirmed by sequencing (Biosune Co., Shanghai) using primers H3-1a and H3-1b. The six isolates with a 440 bp amplicon (A1 to A6) presumed to be *A*. *alternata* (PresA_alt) are FJ1, FJ2, FJ3, HeB3, HeB8, and HeB10 and the six isolates with a 546 bp amplicon (T1 to T6) presumed to be A. *tenuissima* (PreA_ten) are HN5, NMG11, FJ7, HeB4, HeB6, and HeB11.

### Morphological characterization of *Alternaria* species

The same twelve isolates of PresA_alt and PresA_ten were investigated for additional morphological and molecular characteristics. Morphological characters included colony color, growth rate, conidia size, number of septa, and number and shape of conidiophore. Isolates were grown on PDA medium in the dark for 7 d before colony color was recorded and growth rate/day was calculated. Morphology of conidia and conidiophore was observed using the sellotape technique. Briefly, the isolates were grown on synthetic low nutrient agar medium (SNA, including 1g/l KH_2_PO_4_, 1g/l KNO_3_, 0.5g/l KCl, 0.5g/l MgSO_4_·7H_2_O, 0.2g/l glucose, 0.2g/l sucrose and 15g/l agar) [21] in 12 h/12 h periods of light/dark. After 7 d incubation, conidia and conidiophore were adhered to sellotape, and observed under a microscope (Eclipse E100, NIKON). Conidia size and number of septa was averaged from 50 conidia.

### Molecular genetic differentiation of *Alternaria* species

Internal transcribed spacer region (ITS) and partial sequences of glyceraldehyde-3-phosphate dehydrogenase (*GPDH*), translation elongation factor 1-alpha (*TEF1*), β-tubulin, plasma membrane ATPase (*ATPase*), and calmodulin were amplified to investigate variation among *Alternaria* isolates. PCR amplification was performed the same as the components for histone H3 gene except the primers. The primers for ITS and other 6 gene fragments are shown in S1 Table. All PCR amplifications were conducted in 32 cycle repeats with an initial denaturation at 95°C for 4 min and final extension at 72°C for 7 min. The program of 32 cycles for each amplification is shown in S2 Table.

### Data analyses

Sequence alignments were performed using Muscle algorithm [22] implemented in MEGA5 [23]. Histone H3 sequences of PresA_alt and PresA_ten isolates were compared to reference sequence Accession number XP_018380551.1.

The phylogenetic analyses for individual and concatenated dataset of ITS region and 3 gene fragments including β-tubulin, *ATPase* and calmodulin were conducted using Bayesian inference (BI) implemented in MrBayes 3.2.5 [24] after nuleotide substitution saturation test by DAMEB [25]. Dataset of *GPDH* and *TEF1* fragments were not used for phylogenetic analyses because they were all identical in 12 isolates. Concordance among datasets for concatenation was evaluated with the partition homogeneity test (PHT) implemented in PAUP 4.0b10 [26]. The best-fit model for each dataset was determined by JModeltest [27] which was K80 for ITS; HKY for β-tubulin and *ATPase*; HKY+I for calmodulin. Markov chains were run for 2,000,000 generations and sampled every 100 generations. Chain stationary and run parameter convergence were checked using TRACER 1.6 and the first 25% of the convergence runs were discarded as burn-in. The Bayesian consensus tree was generated with 50% majority rule and visualized in FigTree 1.4.3.

The association of molecular marker and *Alternaria* groups was evaluated by phylogeny–trait association analysis using BaTS 2.0 [28] in which association index (AI), parsimony score (PS), and maximum monophyletic clade (MC) were calculated. Statistical significance of trait association was determined by comparing the median posterior estimate for null distribution trees (n = 100, randomly generated) to that for observed values in which *p* values for all three statistics smaller than 0.05 were considered significant association.

## Results

### Identification of *Alternaria* species base on histone H3 gene

**Fig 1:**
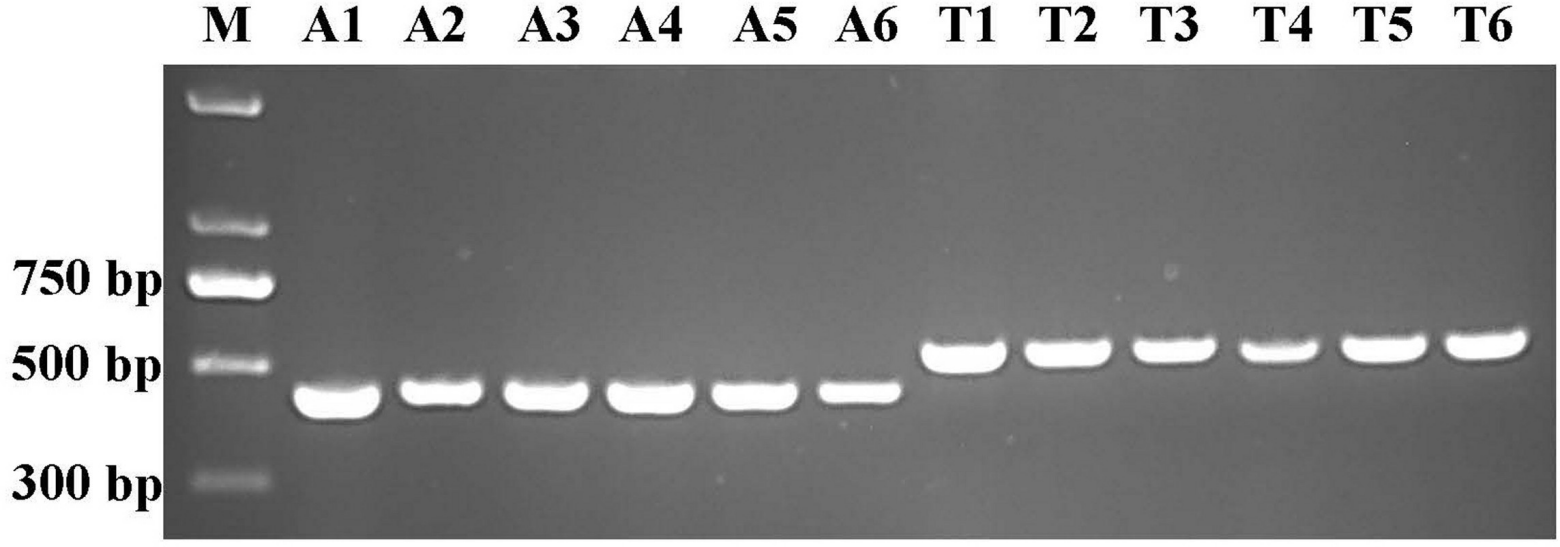
Gel electrophoresis analysis of histone H3 gene amplicons from PresA_alt and PresA_ten isolates with primers H3-1a and H3-1b. M = GeneMarker 2 kb plus DNA ladder (Genstar); A1 to A6 are PresA_alt isolates of FJ1, FJ2, FJ3, HeB3, HeB8 and HeB10, respectively; T1 to T6 are PresA_ten isolates of HN5, NMG11, FJ7, HeB4, HeB6 and HeB11, respectively.

Based on a previously published method [10] to identify *Alternaria* species from potato, 11 and 68 of the 79 isolates from 8 provinces in China are likely to be *A*. *alternata* (PresA_alt) and A. *tenuissima* (PresA_ten), respectively. No isolates with amplicon corresponding to *A*. *solani* were observed (Table 1). PresA_ten isolates dominated in 7 out of 8 locations with one exception in Fujian province where PresA_alt isolates were more frequently observed. Six isolates of each PresA_alt and PresA_ten were randomly selected for further investigation (Fig 1).

### Histone H3 sequence comparison of PresA_alt and PresA_ten isolates

**Fig 2:**
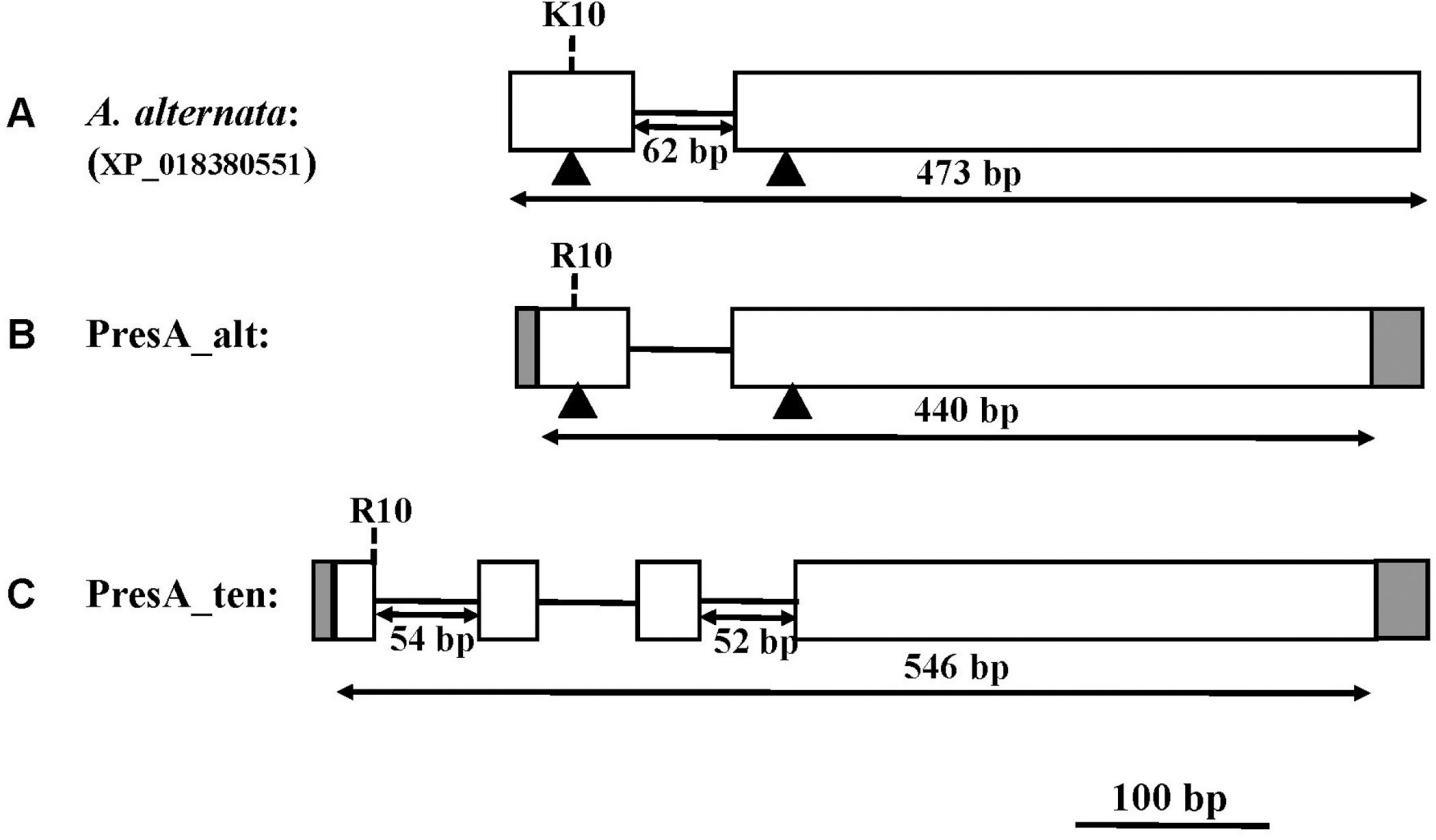
Schematic of histone H3 gene in PresA_alt and PresA_ten isolates. Solid lines indicate introns, blank and gray rectangles indicate cloned and noncloned exons, respectively. Intron and noncloned exon sequences were inferred from *Alternaria alternata* isolate retrieved from GenBank (accession number: XP_018380551, Fig 2A). The vertical dotted line shows the estimated location of the amino acid variation identified in reference isolate, PresA_alt and PresA_ten isolates. The difference in histone amplicon of PresA_ten isolates compared to PresA_alt isolates was showed in Fig 2C as first and third introns whose corresponding positions were indicated by the black triangle in Fig 2A and 2B.

The full length sequence of the histone H3 gene of our reference *A*. *alternata* isolate (Accession number: XP_018380551.1) was 473 bp in length and contained one intron 52 bp. The histone H3 gene sequence fragment amplified with primers H3-1a and H3-1b from six PresA_alt isolates was 440 bp in length and the sequence matched that of the reference isolates. The same fragment amplified from six PresA_ten isolates was 546 bp in length and contained two additional introns 54 bp and 52 bp in size (Fig 2). The deduced amino acid (AA) sequence was identical for all isolates, except for one AA change at position 10. At that position arginine (R) was found in all PresA_alt and PresA_ten isolates instead of lysine (K) for the reference isolate (Fig 2).

### Morphological characterization of PresA_alt and PresA_ten isolates

**Fig 3:**
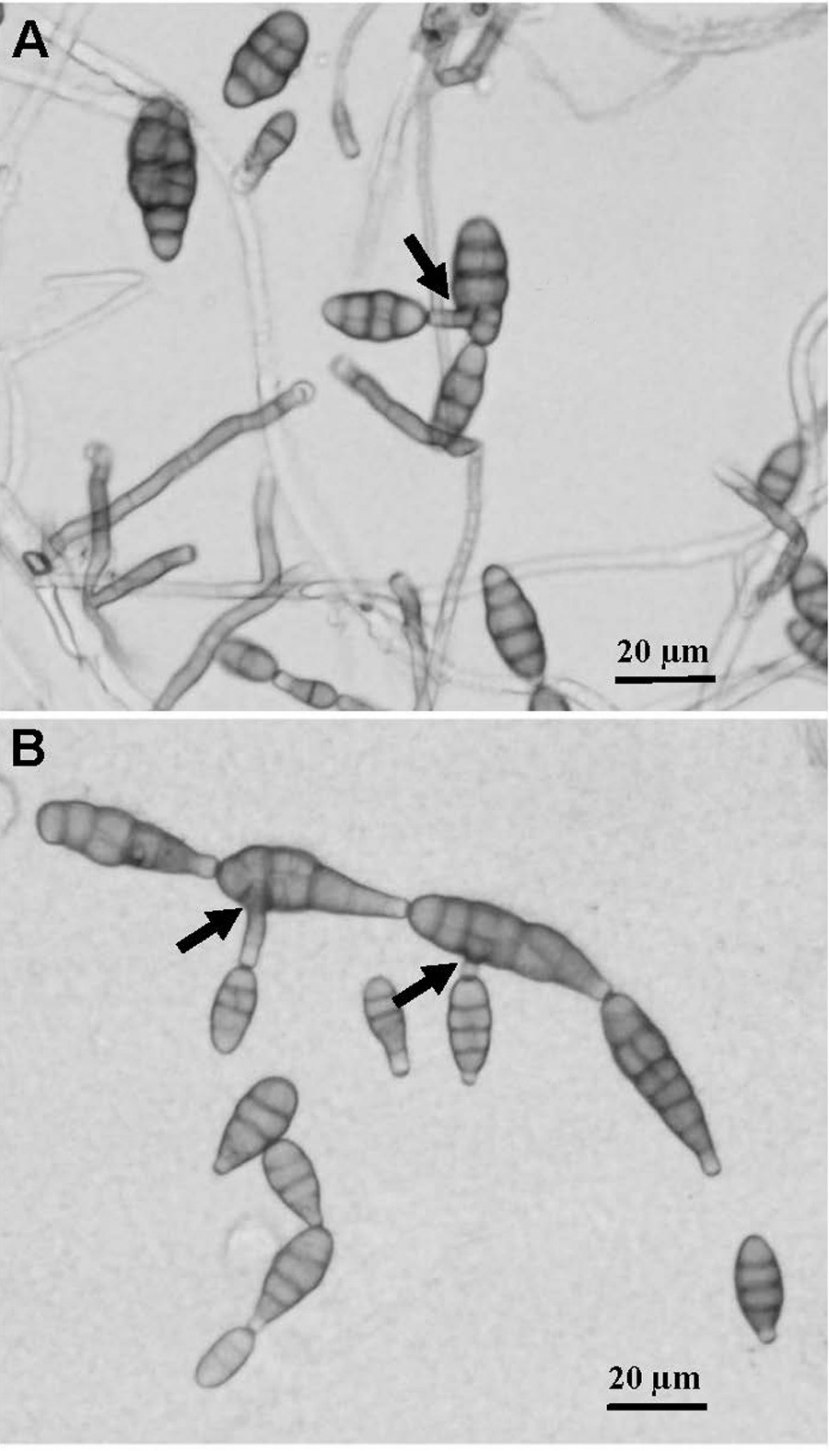
Conidia morphology and branching sporulation of PresA_alt (A) and PresA_ten (B) isolates. The arrow showed the branching sporulation.

The color of colonies on PDA medium varied both among PresA_alt and PresA_ten isolates (Table 2 and S1 Fig). Growth rate for PresA_alt and PresA_ten isolates were ranged from 0.56 to 0.94 and from 0.54 to 0.90, respectively, with no significant difference between those two groups. Similar conidia size and similar number of septa were also observed between PresA_alt and PresA_ten isolates (Table 2). Conidia were obclavate or long ellipsoid in both PresA_alt and PresA_ten isolates. Sporulation in branched chains were observed in four of each PresA_alt and PresA_ten isolates (Table 2 and Fig 3). This was our first indication that the 12 isolates may belong to the same species and that differences in intron numbers in the histone H3 gene may just be natural genetic variation.

**Table 2:** Phenotypic characteristics of PresA_alt and PresA_ten isolates.

		Colony colour	Branching Sporulation^y^	Growth rate (cm/d)	Conidia size (μm)		No. of septa	
Group	Isolate				Length	Width	Transversal	Longitudinal
PresA_alt	A1	Ivory	-	0.76	21.59	10.08	2.2	0.5
	A2	white-green	+	0.94	22.27	11.47	2.2	0.8
	A3	Ivory	+	0.74	19.29	10.77	1.8	0.8
	A4	Ivory	-	0.56	20.25	11.93	1.9	1.0
	A5	Ivory	+	0.77	21.39	10.52	2.0	0.7
	A6	dark green	+	0.85	21.25	11.77	1.9	1.0
PresA_ten	T1	Ivory	+	0.86	20.46	11.41	1.9	0.9
	T2	ivory-brown	+	0.54	19.69	12.29	1.9	1.0
	T3	white-green	-	0.88	23.28	11.43	2.3	0.7
	T4	ivory-brown	-	0.57	21.04	10.34	2.0	0.5
	T5	white-green	+	0.90	23.18	10.73	2.3	0.6
	T6	Ivory	+	0.71	22.67	10.49	2.3	0.6
Mean of PresA_alt isolate				0.77a^z^	21.01a	11.09 a	2.0a	0.8a
Mean of PresA_ten isolate				0.74a	21.72a	11.12a	2.1a	0.7a

^**y**^ Symbol of + and–indicating sporulation in branch was observed and not observed, respectively.

^z^ Values followed by the same letter within a column for PresA_alt and PresA_ten isolate group are not significantly different according to student t test at *p* = 0.05.

### Molecular genetic differentiation of PresA_alt and PresA_ten isolates

**Fig 4:**
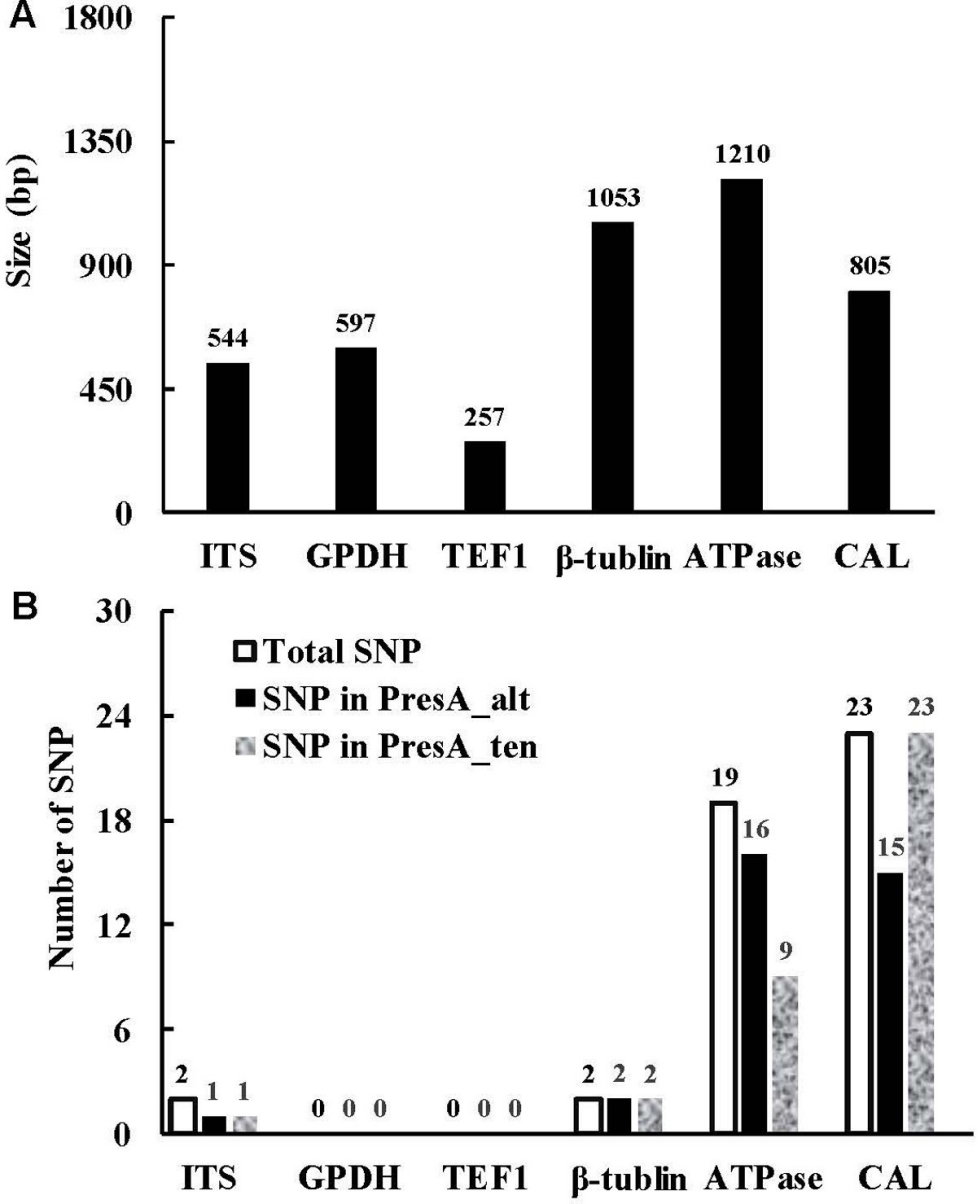
Amplicon size (A) and single nucleotide polymorphism (SNP) (B) in six DNA fragments. ITS, *GPDH*, *TEF1*, *ATPase* and *Cal* were represented internal transcribed spacer region, glyceraldehyde-3-phosphate dehydrogenase, translation elongation factor 1-alpha, plasma membrane ATPase and calmodulin gene, respectively.

The ITS region, *GPDH*, *TEF1*, β-tubulin, *ATPase* and calmodulin sequences were 544 bp, 597 bp, 257 bp, 492 bp, 1210 bp and 805 bp in length, respectively (Fig 4A). The sequences of *GPDH* and *TEF1* were identical for all 12 PresA_alt and PresA_ten isolates; two SNPs were found in both the ITS region and the β-tubulin gene but none was specific for either isolate group; 19 and 26 SNPs were found in the *ATPase* and the calmodulin genes, respectively, and again none of the mutations was specific for the two groups (Fig 4B and S3 Table).

### Phylogenetic analyses of PresA_alt and PresA_ten isolates

**Fig 5:**
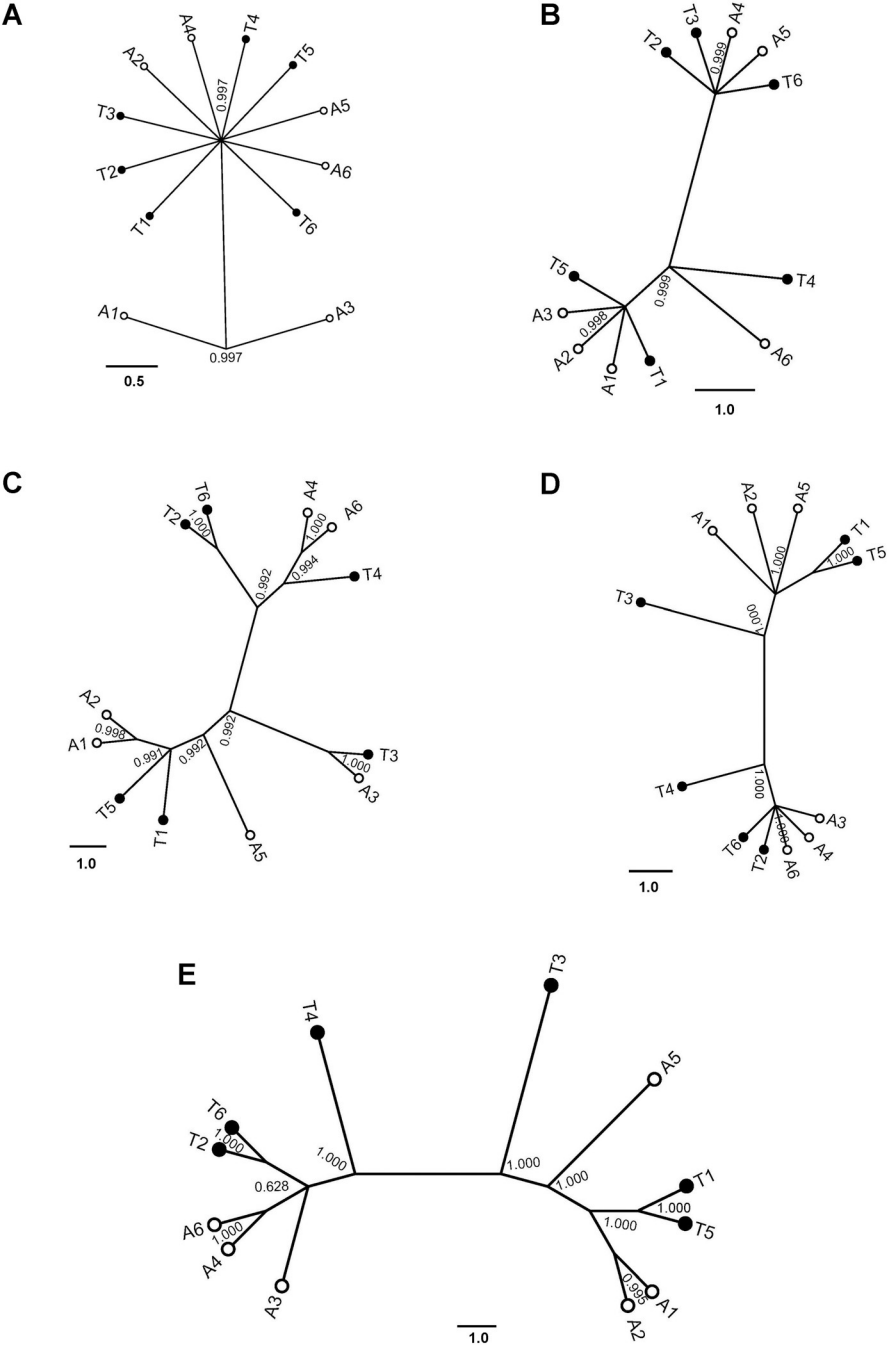
Bayesian 50% majority rule consensus tree base on the DNA sequences in PresA_alt and PresA_ten isolates. The Bayesian posterior probabilities are showed at the nodes. White and black dot represent PresA_alt and PresA_ten isolates, respectively. A-E was constructed on individual ITS (A), β-tubulin (B), *ATPas*e (C) and calmodulin (D) sequence; E was constructed on combined sequences of above 4 sequences.

*GPDH* and *TEF1* gene sequences were not included in the phylogenetic analyses due to identical sequences for all 6 PresA_alt and 6 PresA_ten isolates. For the remaining five loci, all *I*_ss_ (indicator of substitution saturation) were significantly smaller than *I*_ss.c_ (the critical *I*_ss_ values at which the sequences will begin to fail to recover the true tree) indicating little nucleotide substitution saturation (Table 3). PresA_alt and PresA_ten isolates were not in separate clades based on the phylogenetic tree of ITS regions or any of the other four gene sequences. Only two PresA_alt isolates clustered together in the phylogenetic tree based on the ITS region (Fig 5A); PresA_alt and PresA_ten isolates were intermixed based on β-tubulin, *ATPase* and calmodulin gene sequences (Fig 5B–5D). The PresA_alt and PresA_ten isolates were also not separated when all four gene loci were combined (Fig 5E). The association of the two groups (i.e. *Alternaria* species) and phylogeny with each individual molecular marker or combined marker were all rejected with three statistic evaluations in which two at least were greater than 0.05 (AI > 0.05, PI > 0.05, MC > 0.05; Table 4).

**Table 3:** Saturation statistics of nucleotide substitution for all sites in four individual and one combined sequence.

Locus	*I*_ss_	*I*_ss.c_	*p* value^y^
ITS	0.003	0.805	0.000
β-tubulin	0.002	0.825	0.000
*ATPase*	0.006	0.786	0.000
Calmodulin	0.021	0.793	0.000
Combined^z^	0.006	0.827	0.000

^y^*p* value smaller than 0.01 when *I*_ss_ (the indicator of substitution saturation) smaller than *I*_ss.c_ (the critical Iss, at which the sequences will begin to fail to recover the true tree). indicates little to no saturation.

^z^ indicated the sequence were combined by ITS, β-tubulin, *ATPase* and calmodulin.

**Table 4:** Test of *Alternaria* species and phylogeny association for four individual sequences and one combined sequence.

Locus/Gene	Statistic^y^	No. of isolate	Observed mean (95% HPD)	Null mean (95% HPD)	Significance
ITS	AI		0.62 (0.20, 1.02)	0.76 (0.59, 0.91)	0.38
	PS		3.96 (3.00, 5.00)	4.31 (3.85, 4.67)	0.43
	MC(PresA_alt)	6	2.24 (2.00, 4.00)	1.97 (1.67, 2.30)	0.48
	MC(PresA_ten)	6	2.06 (1.00, 3.00)	1.98 (1.66, 2.31)	0.48
β-tubulin	AI		0.94 (0.45, 1.45)	0.75 (0.60, 0.94)	1.00
	PS		4.84 (4.00, 6.00)	4.22 (3.70, 4.85)	1.00
	MC(PresA_alt)	6	1.67 (1.00, 3.00)	2.05 (1.66, 2.36)	0.30
	MC(PresA_ten)	6	1.66 (1.00, 3.00)	1.97 (1.66, 2.36)	0.30
*ATPase*	AI		0.47 (0.43, 0.54)	0.81 (0.35, 1.39)	0.10
	PS		4.00 (4.00, 4.00)	4.40 (3.00, 6.00)	0.41
	MC(PresA_alt)	6	2.00 (2.00, 2.00)	1.93 (1.00, 2.98)	0.55
	MC(PresA_ten)	6	2.00 (2.00, 2.00)	1.99 (1.00, 2.99)	0.52
Calmodulin	AI		0.45 (0.11, 0.73)	0.70 (0.44, 0.90)	0.12
	PS		3.68 (2.00, 5.00)	4.26 (3.00, 5.15)	0.47
	MC(PresA_alt)	6	2.65 (2.00, 3.00)	1.88 (1.31, 2.60)	0.01
	MC(PresA_ten)	6	2.00 (2.00, 2.00)	1.87 (1.17, 2.60)	0.40
Combined^z^	AI		0.23 (0.21, 0.23)	0.78 (0.31, 1.24)	0.05
	PS		3.98 (4.00, 4.00)	4.51 (3.00, 6.00)	0.32
	MC(PresA_alt)	6	2.02 (2.00, 2.00)	1.79 (1.00, 2.06)	0.38
	MC(PresA_ten)	6	2.00 (2.00, 2.00)	1.73 (1.00, 2.92)	0.36

^y^AI, PS, MC and HPD represents association index, parsimony score, maximum monophyletic clade, and highest probability density interval, respectively.

^z^Indicated the sequence were combined by ITS, β-tubulin, *ATPase* and calmodulin.

## Discussion

We erroneously identified two species in our collection of 79 isolates from major potato growing areas of China, based on a previously published method that uses histone H3 gene length for species distinction [10]. Our analysis of morphological features and various gene loci commonly used for fungal species distinction revealed that isolates presumed to *A*. *alternata* and *A*. *tenuissima* were indeed indistinguishable.

*A*.*tenuissima* can be distinguished from *A*. *alternata* based on key morphological features. *A*.*tenuissima* conidia are generally long and consist of unbranched chains whereas *A*. *alternata* produces distinct secondary conidiophores [29]. In our observation, PresA_ten isolates produced for this species atypical branched conidia chains (Fig 3), which indicated those isolates were not *A*. *tenuissima* but more likely *A*. *alternata*. In addition, no other differences in morphological features were found between the PresA_alt and PresA_ten isolates from this study (Table 2).

Due to the difficulties of using morphological features for the identification of species, molecular techniques have been used widely to help dissect species. Neither of the six markers we used differentiated the PresA_alt from PresA_ten isolates. The *GPDH* and *TEF1* genes are housekeeping genes that are generally preserved within species and were used to differentiate species of many genera [30,31]. For example, *GPDH* sequences were used to identify *Alternaria* species [32], *Curvularia inaequalis* and *Bipolaris spicifera* [33]. *TEF1* sequences were used to identify *Macrophomina phaseolina* [34] or used together with other genes [35]. PresA_ten isolates did not cluster apart from PresA_alt isolates in phylogenetic trees based on ITS region, β-tubulin, *ATPase* or calmodulin genes. Although ribosomal ITS sequences proved difficult for use to separate the small-spored *Alternaria* species [7,10], it provided basic information for the identification of our species. In fact, only two PresA_alt and one PresA_ten isolates differed from all others based on a single SNP. In β-tubulin, the same two SNPS were found in each group (S3 Table). Although multiple SNPs and even some deletion were found in *ATPase* and calmodulin genes (Fig 5C and 5D), none were able to cluster the isolates. These molecular results revealed that the PresA_alt and PresA_ten isolates were not genetically distinguishable in key genes.

The combination of gene sequences for phylogenetic tree construction is often more informative compared to the comparison of individual genes. The use of concatenated datasets consisting of rDNA, *TEF-α*, *RBP2* and β-tubulin has been recommended for the study of fungal taxa to improve traditional species concepts [36]. The multigene phylogeny has been applied widely in identification new species [37,38]. Our analysis shows no indication of isolate group separation between PresA_alt and PresA_ten regardless whether single gene sequences or concatenated gene sequences were analyzed (Fig 5E and Table 4).

In this study the molecular basis behind the different-sized histone H3 gene amplicon for PresA_ten and PresA_alt was explored. Two additional introns were observed in PresA_ten isolates resulting in larger amplicons; however, no difference in AA sequence was evident between any of the isolates used in this study to represent PresA_ten and PresA_alt. Therefore we conclude that the observed gene size differences due to intron insertions are simply part of intraspecies variations. Histone H3 variants are common in eukaryotic cells [39–41]. Especially gains and losses of introns are widely observed both within and among species during the evolutionary process [42–44]. The mechanism for intron gain and loss remains unknown, but diverse mechanisms for intron gain were proposed such as genomic duplication, transposable element insertion and mutations resulting in the creation of functional splicing sites and hence occurrence of new introns [45–47]. The major mechanism for intron loss is thought to be recombination of a gene copy with a homologous transcribed RNA transcript [45].

Alignment of the histone H3 gene sequences identified in this study with sequences from the American Type Culture Collection (ATCC) verified that size differences are part of intraspecific variations. PresA_alt-type histone H3 sequence was found in *A*. *alternata* isolate ATCC 66892 (sequence number: AA2CTG00204) and PresA_ten-type histone H3 sequence was found in two other *A*. *alternata* isolates, ATCC 11680 and ATCC 66891 (sequence number: ATNCTG00656 and AATCTG00004, respectively). In addition, PresA_alt-type histone H3 sequence was found in *A*. *tenuissima* isolate ATCC 96828 (sequence number: AT2CTG00436). ATCC 66892, ATCC 66892 and ATCC 96828 were also identified by E.G. Simmons and designated EGS 34–016, EGS 34–039 and EGS 34–015, respectively [48].

We conclude that the histone H3 gene is not suitable to distinguish *A*. *tenuissima* from *A*. *alternata* and that the former species was likely overestimated in previous studies [10]. Morphological traits are still the most important basis in identifying *Alternaria* species in potato currently, and the presA_ten isolates identified by histone H3 gene were likely a differentiate genotype of *A*. *alternata*.

## Supporting information

S1 Table Primers used in this study. (DOCX)

S2 Table PCR settings of 32 cycles for amplification of the ITS region and six other gene fragments. (DOCX)

S3 Table Single nucleotide polymorsim (SNP) in ITS region, β-tubulin, plasma membrane ATPase (*ATPase*), and calmodulin. (XLS)

S1 Fig Colony morphology of PresA_alt (A-C) and PresA_ten (D-F) isolates. A-C was the colony of A2, A5 and A6 isolate, respectively; D-F was the colony of T2, T5 and T6 isolate, respectively.
(JPG)

S1 Raw image The file showed the original image of Fig 1. (PDF)

## References

[1] Tsedaley B (2014) Review on early blight (Alternaria spp.) of potato disease and its management options. J Biol Agric Healthc 4: 191–198.

[2] Stevenson WR, James RV, Inglis DA, Johnson DA, Schotzko RT, et al (2007) Fungicide spray programs for defender, a new potato cultivar with resistance to late blight and early blight. Plant Dis 91: 1327–1336. 10.1094/PDIS-91-10-1327 30780516

[3] Kirk W, Wharton P (2012) Brown leaf spot Mich. Ext. Bull. E-3182 Department of Plant, Soil and Microbial Science, Michigan State University, East Lansing, MI.

[4] Jones LR, Grout AJ (1997) Notes on two species of *Alternaria*. J Torrey Bot Soc 24: 254–258.

[5] Ardestani ST, Sharifnabi B, Zare R, Moghadam AA (2010) New *Alternaria* species associated with potato leaf spot in various potato growing region of Iran. Iran J Plant Pathol 45: 83–86.

[6] Ayad D, Leclerc S, Hamon B, Kedad A, Bouznad Z, et al (2017) First report of early blight caused by *Alternaria protenta* on potato in Algeria. Plant Dis 101: 836–837.

[7] Landschoot S, Vandecasteele M, De Baets B, Hofte M, Audenaert K, et al (2017) Identification of *A*. *arborescens*, *A*. *grandis*, and *A*. *protenta* as new members of the European *Alternaria* population on potato. Fungal Biol 121: 172–188. 10.1016/j.funbio.2016.11.005 28089048

[8] Shoaib A, Akhtar N, Akhtar S, Hafeez R (2014) First report of *Alternaria longipes* causing leaf spot of potato cultivar sante in Pakistan. Plant Dis 98: 1742. 10.1094/PDIS-05-14-0539-PDN 30703895

[9] Tymon LS, Peever TL, Johnson DA (2016) Identification and enumeration of small-spored *Alternaria* species associated with potato in the US Northwest. Plant Dis 100: 465–472. 10.1094/PDIS-03-15-0263-RE 30694153

[10] Zheng HH, Zhao J, Wang TY, Wu XH (2015) Characterization of *Alternaria* species associated with potato foliar diseases in China. Plant Pathol 64: 425–433.

[11] Gannibal PB, Orina AS, Mironenko NV, Levitin MM (2014) Differentiation of the closely related species, *Alternaria solani* and *A*. *tomatophila*, by molecular and morphological features and aggressiveness. Eur J Plant Pathol 139: 609–623.

[12] Tymon L, Johnson DA (2014) Fungicide resistance of two species of *Alternaria* from potato in the Columbia basin of Washington. Plant Dis 98: 1648–1653. 10.1094/PDIS-12-13-1199-RE 30703884

[13] Rueda-Hernandez KR, Cardona AS, Cadavid-Restrepo GE, Benjumea CIS, Gutierrez GPC, et al (2013) Differential organ distribution, pathogenicity and benomyl sensitivity of *Colletotrichum spp*. from blackberry plants in Northern Colombia. J Phytopathol 161: 246–253.

[14] Chen SN, Luo CX, Hu MJ, Schnabel G (2016) Sensitivity of *Colletotrichum* species, including *C*. *fioriniae* and *C*. *nymphaeae*, from peach to demethylation inhibitor fungicides. Plant Dis 100: 2434–2441. 10.1094/PDIS-04-16-0574-RE 30686167

[15] Hu M-J, Grabke A, Dowling ME, Holstein HJ, Schnabel G (2015) Resistance in *Colletotrichum siamense* from peach and blueberry to thiophanate-methyl and azoxystrobin. Plant Dis 99: 806–814. 10.1094/PDIS-10-14-1077-RE 30699530

[16] Peres NAR, Souza NL, Zitko SE, Timmer LW (2002) Activity of benomyl for control of postbloom fruit drop of citrus caused by *Colletotrichum acutatum*. Plant Dis 86: 620–624. 10.1094/PDIS.2002.86.6.620 30823234

[17] Ishii H, Zhen F, Hu M, Li X, Schnabel G (2016) Efficacy of SDHI fungicides, including benzovindiflupyr, against *Colletotrichum* species. Pest Manag Sci 72: 1844–1853. 10.1002/ps.4216 26732510

[18] Thomma B (2003) *Alternaria* spp.: from general saprophyte to specific parasite. Mol Plant Pathol 4: 225–236. 10.1046/j.1364-3703.2003.00173.x 20569383

[19] Chen FP, Fan JR, Zhou T, Liu XL, Liu JL, et al (2012) Baseline sensitivity of *Monilinia fructicola* from China to the DMI fungicide SYP-Z048 and analysis of mutants. Plant Dis 96: 416–422. 10.1094/PDIS-06-11-0495 30727143

[20] Glass NL, Donaldson GC (1995) Development of primer sets designed for use with the PCR to amplify conserved genes from filamentous ascomycetes. Appl Environ Microbiol 61: 1323–1330. 7747954 10.1128/aem.61.4.1323-1330.1995 PMC167388

[21] Nirenberg HI, Aoki T (1997) *Fusarium nisikadoi*, a new species from Japan. Mycoscience 38: 329–333.

[22] Edgar RC (2004) MUSCLE: multiple sequence alignment with high accuracy and high throughput. Nucleic Acids Res 32: 1792–1797. 10.1093/nar/gkh340 15034147 PMC390337

[23] Tamura K, Stecher G, Peterson D, Filipski A, Kumar S (2013) MEGA6: Molecular evolutionary genetics analysis version 6.0. Mol Biol Evol 30: 2725–2729. 10.1093/molbev/mst197 24132122 PMC3840312

[24] Ronquist F, Teslenko M, van der Mark P, Ayres DL, Darling A, et al (2012) MrBayes 3.2: Efficient Bayesian phylogenetic inference and model choice across a large model space. Syst Biol 61: 539–542. 10.1093/sysbio/sys029 22357727 PMC3329765

[25] Xia X (2017) DAMBE6: new tools for microbial genomics, phylogenetics, and molecular evolution. J Hered 108: 431–437. 10.1093/jhered/esx033 28379490 PMC5434544

[26] Swofford DL (2002) PAUP*: Phylogenetic analysis using parsimony (*and other methods), Version 4. Sinauer Associates; Sunderland, MA.

[27] Nylander JAA (2008) MrModeltest v2.3. Program distributed by the author. Evolutionary Biology Centre, Uppsala University.

[28] Parker J, Rambaut A, Pybus OG (2008) Correlating viral phenotypes with phylogeny: accounting for phylogenetic uncertainty. Infect Genet Evol 8: 239–246. 10.1016/j.meegid.2007.08.001 17921073

[29] Simmons EG (2007) *Alternaria*: an identification manual. CBS Biodiversity Series. 6:1–775.

[30] Kiss N, Homa M, Manikandan P, Mythili A, Krizsan K, et al (2020) New species of the genus Curvularia: C. tamilnaduensis and C. coimbatorensis from fungal Keratitis cases in South India. Pathogens 9. 10.3390/pathogens9010009 PMC7168623 31861831

[31] Heidari K, Mehrabi-Koushki M, Farokhinejad R (2018) *Curvularia mosaddeghii* sp nov., a novel species from the family Pleosporaceae. Mycosphere 9: 635–646.

[32] Jaouani A, Neifar M, Prigione V, Ayari A, Sbissi I, et al (2014) Diversity and enzymatic profiling of halotolerant micromycetes from Sebkha El Melah, a Saharan salt flat in Southern Tunisia. Biomed Res Int 2014: 439197 10.1155/2014/439197 25136587 PMC4124809

[33] Amaradasa BS, Amundsen K (2014) First report of *Curvularia inaequalis* and *Bipolaris spicifera* causing leaf blight of buffalograss in Nebraska. Plant Dis 98: 279–279. 10.1094/PDIS-05-13-0487-PDN 30708754

[34] Sousa ES, Melo MP, Pires LL, Silva BA, Garcia MEM, et al (2017) First report of Macrophomina phaseolina causing charcoal rot in lima bean (Phaseolus lunatus) in Brazil. Plant Dis 101: 1551–1551.

[35] de Hoog GS, Nishikaku AS, Fernandez-Zeppenfeldt G, Padin-Gonzalez C, Burger E, et al (2007) Molecular analysis and pathogenicity of the *Cladophialophora carrionii* complex, with the description of a novel species. Stud Mycol 58: 219–234. 10.3114/sim.2007.58.08 18491001 PMC2104744

[36] Hyde KD, Hongsanan S, Jeewon R, Bhat DJ, McKenzie EHC, et al (2016) Fungal diversity notes 367–490: taxonomic and phylogenetic contributions to fungal taxa. Fungal Divers 80: 1–270.

[37] Pem D, Hyde KD, Doilom M, Camporesi E, Hongsanan S, et al (2019) Multigene phylogenetic analyses to establish new *Valsaria* species and taxonomic significance of spore ornamentation. PLoS ONE 14: e0217982 10.1371/journal.pone.0217982 31242234 PMC6594670

[38] Kawai H, Akita S, Hashimoto K, Hanyuda T (2020) A multigene molecular phylogeny of Eisenia reveals evidence for a new species, Eisenia nipponica (Laminariales), from Japan. Eur J Phycol. 10.1080/09670262.2019.1692911

[39] Tang MCW, Jacobs SA, Mattiske DM, Soh YM, Graham AN, et al (2015) Contribution of the two genes encoding histone variant H3.3 to viability and fertility in mice. PLoS Genet 11. 10.1371/journal.pgen.1004964 PMC4335506 25675407

[40] Allis CD, Glover CV, Gorovsky MA (1979) Micronuclei of *Tetrahymena* contain two types of histone H3. Proc Natl Acad Sci USA 76: 4857–4861. 10.1073/pnas.76.10.4857 291904 PMC413036

[41] Lee HR, Zhang WL, Langdon T, Jin WW, Yan HH, et al (2005) Chromatin immunoprecipitation cloning reveals rapid evolutionary patterns of centromeric DNA in *Oryza* species. Proc Natl Acad Sci USA 102: 11793–11798. 10.1073/pnas.0503863102 16040802 PMC1187982

[42] Zhang L-Y, Yang Y-F, Niu D-K (2010) Evaluation of models of the mechanisms underlying intron loss and gain in *Aspergillus* fungi. J Mol Evol 71: 364–373. 10.1007/s00239-010-9391-6 20862581

[43] Milia G, Camiolo S, Avesani L, Porceddu A (2015) The dynamic loss and gain of introns during the evolution of the *Brassicaceae*. Plant J 82: 915–924. 10.1111/tpj.12860 25899207

[44] Croll D, McDonald BA (2012) Intron gains and losses in the evolution of *Fusarium* and *Cryptococcus* fungi. Genome Biol Evol 4: 1148–1161. 10.1093/gbe/evs091 23054310 PMC3514964

[45] Roy SW, Irimia M (2009) Mystery of intron gain: new data and new models. Trends Genet 25: 67–73. 10.1016/j.tig.2008.11.004 19070397

[46] Roy SW (2009) Intronization, de-intronization and intron sliding are rare in *Cryptococcus*. BMC Evol Biol 9: 192 10.1186/1471-2148-9-192 19664208 PMC2740785

[47] Koonin EV (2009) Intron-dominated genomes of early ancestors of eukaryotes. J Hered 100: 618–623. 10.1093/jhered/esp056 19617525 PMC2877545

[48] Dang HX, Pryor B, Peever T, Lawrence CB (2015) The *Alternaria* genomes database: a comprehensive resource for a fungal genus comprised of saprophytes, plant pathogens, and allergenic species. BMC Genomics 16. 10.1186/s12864-015-1430-7 PMC4387663 25887485

