# Correction: Histone H3 gene is not a suitable marker to distinguish *Alternaria tenuissima* from *A*. *alternata* affecting potato

**DOI:** 10.1371/journal.pone.0273605

**Published:** 2022-08-22

**Authors:** Yue Zhang, Peiyu Tian, Guohua Duan, Fangluan Gao, Guido Schnabel, Jiasui Zhan, Fengping Chen

After the publication of [1] it was noticed that the minimal dataset for the sequencing results is missing. The authors provide the sequences of genes used to identify the 12 isolates under GenBank numbers ON413681 to ON413692 (ITS), ON442359 to ON442370 (plasma membrane ATPase), ON442371 to ON442382 (calmodulin), ON442383 (GAPDH), ON442384 (TEF1), ON442385 to ON442396 (beta-tubulin), and ON442397 to ON442408 (histone H3). The sequences for TEF1 and GAPDH were submitted only once, because these two loci are identical in the 12 isolates. With this correction, all relevant data for [1] are now provided.

In the Data Analysis section of the Material and Methods, there is an error in the second sentence of the first paragraph. The correct sentence is: Histone H3 sequences of PresA_alt and PresA_ten isolates were compared to reference sequence Accession number NW_017306216.1. This is the genomic scaffold of *A*. *alternata* and contains two histone-fold-containing proteins.
