# Supplementary material for: Histone H3 gene is not a suitable marker to distinguish Alternaria tenuissima from A. alternata affecting potato
Source: PLoS One. 2020 Apr 23;15(4):e0231961. doi: 10.1371/journal.pone.0231961 (PMC7179870; doi:10.1371/journal.pone.0231961)
Supplement: S1 Table — (DOCX) [file pone.0231961.s001.docx]

Table S1 Primers used in this study

| Primer | Sequence (5'-3') | Function | Origin |
| --- | --- | --- | --- |
| H3-1a | ACTAAGCAGACCGCCCGCAGG | Amplification and sequencing partial histone H3 gene | ([Glass and Donaldson 1995](#_ENREF_3)) |
| H3-1b | GCGGGCGAGCTGGATGTCCTT |  |  |
| ITS1 | TCCGTAGGTGAACCTGCGG | Amplification and sequencing Internal transcribed spacer region (ITS) | ([White et al. 1990](#_ENREF_7)) |
| ITS4 | TCCTCCGCTTATTGATATGC |  |  |
| Gpd_F | CAACGGCTTCGGTCGCATTG | Amplification and sequencing partial glyceraldehyde-3-phosphate dehydrogenase (*GPDH*) gene | ([Berbee et al. 1999](#_ENREF_1)) |
| Gpd_R | GCCAAGCAGTTGGTTGTGC |  |  |
| TEF1_F | CATCGAGAAGTTCGAGAAGG | Amplification and sequencing partial translation elongation factor 1-alpha (*TEF1*) gene | ([Carbone and Kohn 1999](#_ENREF_2)) |
| TEF1_R | TACTTGAAGGAACCCTTACC |  |  |
| Tub_F2 | CAGCTCGAGCGTATGAACGTCT | Amplification and sequencing partial β-tubulin gene | ([McKay 1998](#_ENREF_6)) |
| Tub_R2 | TGTACCAATGCAAGAAAGCCTT |  |  |
| ATP_F | ATCGTCTCCATGACCGAGTTCG | Amplification and sequencing partial plasma membrane *ATPase* gene | ([Lawrence et al. 2013](#_ENREF_5)) |
| ATP_R | TCCGATGGAGTTCATGATAGCC |  |  |
| Cal_F1 | AGCAAGTCTCCGAGTTCAAGG | Amplification and sequencing partial calmodulin gene | ([Lawrence et al. 2013](#_ENREF_5)) |
| Cal_R1 | CTTCTGCATCATCAYCTGGACG |  |  |
