# Supplementary material for: Histone H3 gene is not a suitable marker to distinguish Alternaria tenuissima from A. alternata affecting potato
Source: PLoS One. 2020 Apr 23;15(4):e0231961. doi: 10.1371/journal.pone.0231961 (PMC7179870; doi:10.1371/journal.pone.0231961)
Supplement: S2 Table — (DOCX) [file pone.0231961.s002.docx]

Table S2 PCR settings of 32 cycles for amplification of the ITS region and six other gene fragments

| Locus/Gene | Denaturation | Annealing | Extension |
| --- | --- | --- | --- |
| Histone H3 | 95℃ for 30s | 60℃ for 40s | 72℃ for 60s |
| ITS | 95℃ for 30s | 58℃ for 40s | 72℃ for 60s |
| *GPDH* | 95℃ for 30s | 60℃ for 40s | 72℃ for 60s |
| *TEF1* | 95℃ for 30s | 55℃ for 40s | 72℃ for 60s |
| β-tubulin | 95℃ for 30s | 60℃ for 40s | 72℃ for 120s |
| *ATPase* | 95℃ for 30s | 60℃ for 40s | 72℃ for 120s |
| Calmodulin | 95℃ for 30s | 58℃ for 40s | 72℃ for 60s |
