# Supplementary figures and images for: Histone H3 gene is not a suitable marker to distinguish Alternaria tenuissima from A. alternata affecting potato
Source: PLoS One. 2020 Apr 23;15(4):e0231961. doi: 10.1371/journal.pone.0231961 (PMC7179870; doi:10.1371/journal.pone.0231961)

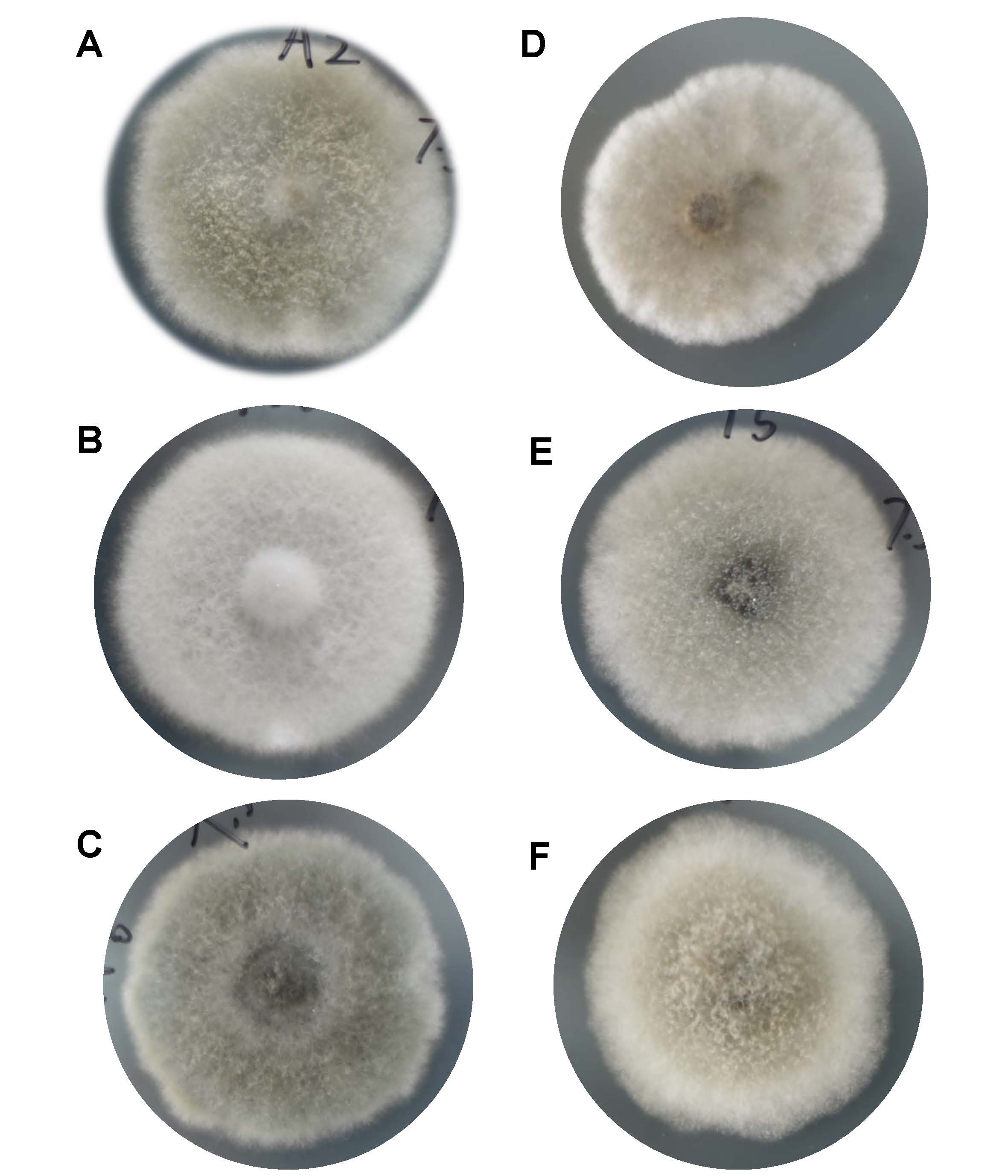

Supplement: S1 Fig — Colony morphology of PresA_alt (A-C) and PresA_ten (D-F) isolates. A-C was the colony of A2, A5 and A6 isolate, respectively; D-F was the colony of T2, T5 and T6 isolate, respectively. (JPG) [file pone.0231961.s004.jpg]
