# Supplementary material for: Histone H3 gene is not a suitable marker to distinguish Alternaria tenuissima from A. alternata affecting potato
Source: PLoS One. 2020 Apr 23;15(4):e0231961. doi: 10.1371/journal.pone.0231961 (PMC7179870; doi:10.1371/journal.pone.0231961)
Supplement: S1 Raw image — (PDF) [file pone.0231961.s005.pdf]

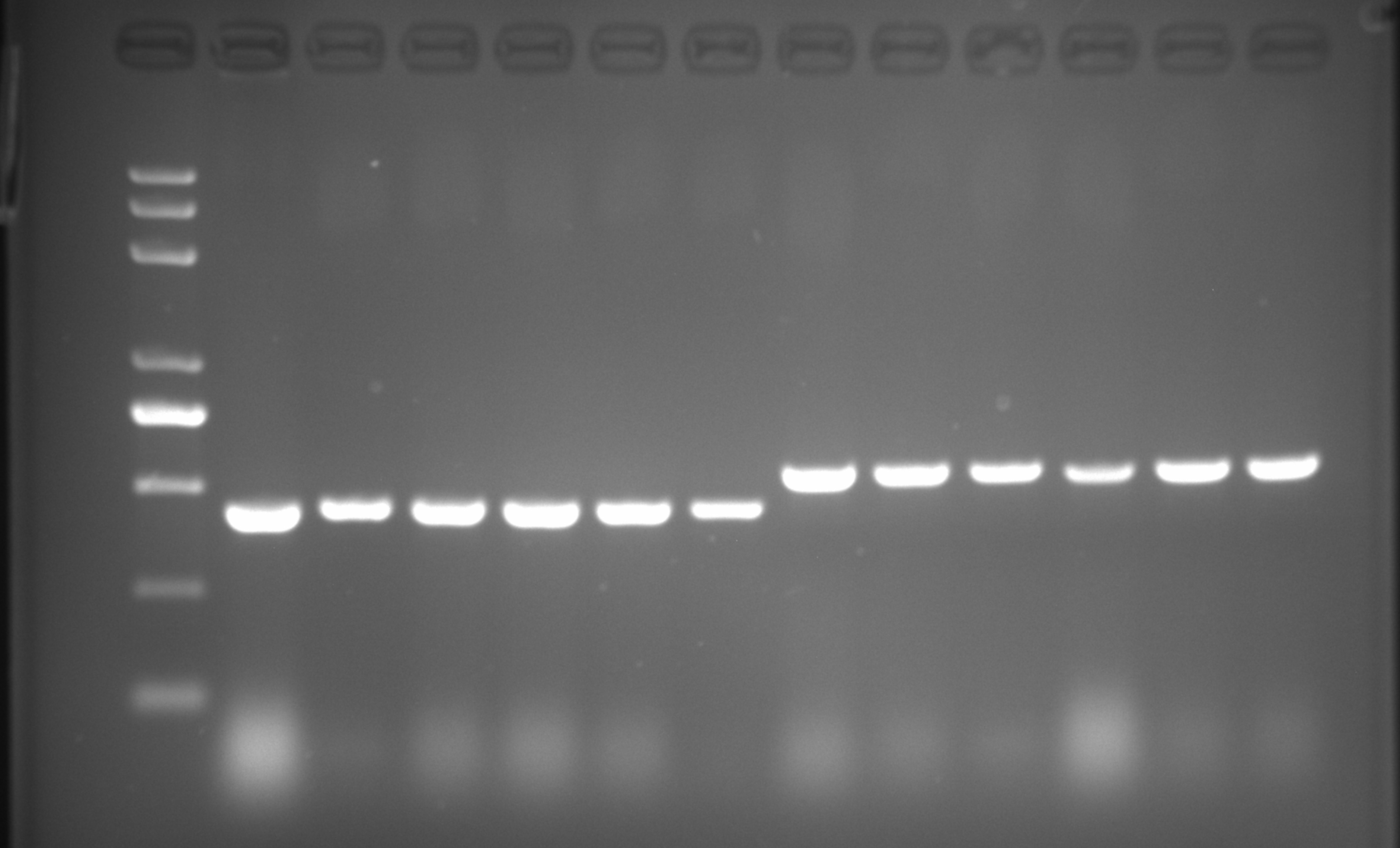

Note: This is the original image of Figure 1, captured by Syngene Gbox-F3 system with GeneSys (1.2.5.0 version, Gene company ltd.) using Auto capture mode. The image showed gel electrophoresis analysis of histone H3 gene amplicons from PresA_alt and PresA_ten isolates with primers H3-1a and H3-1b. Lane 1 was GeneMarker 2 kb plus DNA ladder (Genstar); Lane 2 to 7 were PresA_alt isolates of FJ1, FJ2, FJ3, HeB3, HeB8 and HeB10, respectively; Lane 8 to 13 were PresA_ten isolates of HN5, NMG11, FJ7, HeB4, HeB6 and HeB11, respectively.
